# Multilink communities of multiplex networks

**DOI:** 10.1371/journal.pone.0193821

**Published:** 2018-03-20

**Authors:** Raul J. Mondragon, Jacopo Iacovacci, Ginestra Bianconi

**Affiliations:** 1 School of Electronic Engineering and Computer Science, Queen Mary University of London, London, United Kingdom; 2 School of Mathematical Sciences, Queen Mary University of London, London, United Kingdom; University of Bristol, UNITED KINGDOM

## Abstract

Multiplex networks describe a large number of complex social, biological and transportation networks where a set of nodes is connected by links of different nature and connotation. Here we uncover the rich community structure of multiplex networks by associating a community to each multilink where the multilinks characterize the connections existing between any two nodes of the multiplex network. Our community detection method reveals the rich interplay between the mesoscale structure of the multiplex networks and their multiplexity. For instance some nodes can belong to many layers and few communities while others can belong to few layers but many communities. Moreover the multilink communities can be formed by a different number of relevant layers. These results point out that mesoscopically there can be large differences in the compressibility of multiplex networks.

## Introduction

The current Big Data explosion requires the development of new algorithms and theoretical methods to extract information from large datasets. Often in this context, it is advantageous to combine information coming from different sources and to represent the data by a multiplex network [1–5]. A multiplex network is formed by a set of nodes connected in different layers by links indicating interactions of different types. Multiplex networks are ubiquitous spanning from complex infrastructure networks [4, 6, 7], to social [8–11] biological [8, 12] and transportation networks [13, 14]. For instance, individuals can be related by different type of social ties, neurons can interact through chemical synapses and electrical gap junctions, and two locations can be connected by different means of transportation.

A multiplex network tends to have a richer structure than single networks and this richness is reflected in its communities [9, 10, 15–17]. The communities of a multiplex network cannot be obtained by considering its layers individually. Some communities might exist only in one layer, other communities can overlap on many layers and finally there are communities that only exist when considering the whole structure of the multiplex network. Several algorithms [9, 18–22] have been recently proposed to detect multilayer communities. These include methods based on multilayer modularity optimization [9, 20], diffusion properties on multilayer networks [18, 21] and consensus clustering [22]. All these techniques are node-based community detection methods where each node or each replica-node (realization of a node in a given layer) is classified in one community. Interestingly in the framework of single-layer community detection [23, 24] it has been observed that link-based community detection methods [25, 26] can be very fruitful to describe the mesoscale organization of networks when nodes belong to several communities at the same time [27]. The need to extend the link communities to multiplex network is rather pressing. For instance if we consider individuals interacting through different on-line social network platforms, say Twitter and Facebook, it might be misleading to think that an individual or an account (a Twitter or Facebook account) might belong just to a single community. In fact, influential Twitter of Facebook accounts tend to reach more than one community of the same online platform.

In simple networks any two nodes can be either connected or not connected by a link, in multiplex network any two nodes can be connected in multiple ways. We say that two nodes are connected via a *multilink* [3, 11], where the multilink describes the pattern of connections between two nodes. In this work we propose a multilink community detection method for multiplex networks which extends link communities to the multiplex network framework. Our community detection method is based on the similarity of incident multilinks. In order to reduce unnecessary layer-information, the similarity between two multilinks is measured by comparing the local structure of the multiplex against a local, maximum entropy null model. To avoid introducing bias via the null model, the null model describes our state of knowledge of the multiplex in a way that is maximally noncommittal to the layered structure.

Here we show that using the proposed multilink community detection method not only we are able to extract relevant information on the mesoscale structure of multiplex networks, but also we can contribute to the scientific debate about the compressibility of the multiplex network structures. Recent research on multiplex networks questions whether it is opportune to aggregate or disaggregate their layers. Aggregation of layers could be useful for removing redundant information. De Domenico et al. [28], have shown that for the vast majority of multiplex networks there is trade-off between the information content and the minimization of their total number of layers. The case of disaggregating a single network to a multi-layer network has been considered by Vales-Catala et al. in Ref. [29]. According to their results some single networks are better represented as multiplex networks because they are effectively the result of a blind multiplex network aggregation procedure. Finally, Peixoto [30], using a statistical inference approach, has revealed that there is no clear answer, the benefits of the aggregation or disaggregation of the layers are dependent on the system under study.

Here we show that actually the optimal answer to the question whether it is more appropriate to aggregate or disaggregate a general multiplex network might not be global but mesoscale. Our analysis of social, biological and transportation networks reveals that in multiplex networks there is a very rich interplay between their mesoscale organization and their multiplexity. Multilinks communities can include connections of only one layer or of multiple layers. Additionally we observe that not always the layer activity (in how many layers a node is connected) correlates with the community activity (in how many communities a node can be found). For example there can be nodes that are connected in many layers (high layer activity) but belong only to few multilink communities (low community activity) and nodes belonging to few layers (low layer activity) but belonging to many multilink communities (high community activity). The first possibility suggests that mesoscopically the network could be compressed while the second possibility suggests that mesoscopically the network could be expanded into many layers making a case for a definition of a mesoscale compressibility of the multiplex network.

## Materials and methods

### Multiplex network

Let us consider a multiplex network formed by *N* nodes and *M* layers *α* = 1, 2, …, *M*. The multiplex network is the set of *M* networks $\vec{G}=\left(G^{\left[1\right]},G^{\left[2\right]},\ldots ,G^{\left[\alpha \right]},\ldots ,G^{\left[M\right]}\right)$ where each network *G*^[*α*]^ = (*V*, *E*^[*α*]^) is formed by the same set of *N* nodes *V* = {*i*; *i* = 1, 2, …, *N*} and by the set of links *E*^[*α*]^ which describe the connections in layer *α*. We assume that all these networks are undirected and we represent each layer *α* = 1, 2, …, *M* by the adjacency matrix $a^{\left[\alpha \right]}$. The whole multiplex network can be expressed via its multilinks [3, 11]. Every pair of nodes (*i*, *j*) is connected by a multilink

$$\begin{array}{c} {\vec{m}}_{ij}=\left(m_{ij}^{\left[1\right]},m_{ij}^{\left[2\right]},\ldots ,m_{ij}^{\left[\alpha \right]}\ldots m_{ij}^{\left[M\right]}\right), \end{array}$$ (1)

with $m_{ij}^{\left[\alpha \right]}=a_{ij}^{\left[\alpha \right]}$ indicating in which layers of the multiplex network the two nodes are connected. The vector ${\vec{m}}_{ij}$ specify what we call the *multilink composition*, i.e. in which layers node *i* and node *j* are connected. Whenever node *i* and node *j* are connected at least in one layer, i.e. $\vec{m}\neq \vec{0},$ we say that they are connected by a non-trivial multilink. To decide if a non-trivial multilink exist, it is convenient to construct the aggregated network $\hat{G}$ formed by the *N* nodes of the multiplex. The adjacency matrix **A** of the aggregated network $\hat{G}$ has elements

$$\begin{array}{c} A_{ij}=\theta \left(\sum_{\alpha =1}^{M}a_{ij}^{\left[\alpha \right]}\right), \end{array}$$ (2)

where *θ*(*x*) is the step function *θ*(*x*) = 1 if *x* > 0 and *θ*(*x*) = 0 if *x* ≤ 0. We indicate with *L* = ∑_*i*<*j*_ *A*_*ij*_ the total number of links of the aggregated network, or equivalently the number of non-trivial multilinks.

In a multiplex network the nodes might not be connected in each layer. The number of layers in which a node is connected (or active) is called the *node activity* [8, 31] and reveals relevant coarse grained information about the node.

### Multilink similarity

In the context of single networks several community detection methods use hierarchical clustering applied either to a similarity matrix between nodes [32] or between links [25, 26]. Here we construct a hierarchical clustering of multiplex networks based on a measure of similarity between incident multilinks. By defining the similarity between multilinks here we generalize the link communities previously defined for single layers [25, 26] to multiplex networks.

In a similar spirit to the use of the modularity function for detecting node communities [33], the similarity between incident multilinks is evaluated by comparing simultaneously the cohesiveness and the multiplexity of their neighborhood to a maximum entropy null model.

To every pair of multilinks connecting nodes *i* and *k* and nodes *j* and *s* we assign the similarity *S*_*ik*,*js*_. The similarity *S*_*ik*,*js*_ is non-zero only between incident multilinks (i.e. for *s* = *k*) and is a function of two parameters: *ϵ* and *z*. The parameter *ϵ* ∈ (0, 1) can be tuned depending on the role that we want to assign to the composition of the two incident multilinks with respect to their local neighborhood. The additional parameter *z* ∈ (0, 1) evaluates the role of multiplexity and represent the cost we want to attribute to incident multilinks of different composition.

Specifically the non-zero similarities *S*_*ik*,*jk*_ are given by

$$\begin{array}{c} S_{ik,jk}=\epsilon \sigma_{ijk}+\left(1-\epsilon \right)\sigma_{ij\setminus k}. \end{array}$$ (3)

where *σ*_*ijk*_ evaluates the contribution of the two incident multilinks while *σ*_*ij*\*k*_, evaluates instead the contribution due to the existence of other multilinks, joining node *i* and node *j* directly or by paths of length two excluding node *k*. The parameter *ϵ* ∈ (0, 1) tunes the relative importance between these two contributions.

The term *σ*_*ijk*_ is expressed as

$$\begin{array}{c} \sigma_{ijk}=z^{\beta_{ik,jk}}, \end{array}$$ (4)

with

$$\begin{array}{c} \beta_{ij,rs}=1-\frac{\sum_{\alpha =1}^{M}m_{ij}^{\left[\alpha \right]}m_{rs}^{\left[\alpha \right]}}{M}. \end{array}$$ (5)

The smaller is *z* the larger is the “penalty” imposed to the similarity *S*_*ik*,*jk*_ between non-zero incident multilinks if node *i* and node *j* are connected in layers different from the one connecting node *j* and *k*. Therefore *z* tunes the layer dependance of the multilink communities. A large value of *z* favors communities existing only in one layer or overlapping in different layers, while a smaller value of *z* allows for multilink communities of multilinks with different composition. If the multilinks connecting nodes (*i*, *k*) and (*j*, *k*) have not even a link in a common layer then *β*_*ik*,*jk*_ = 1 and $z^{\beta_{ik,jk}}=z$, indicating the maximum cost attributed to multiplexity. If, on the contrary the two multilinks have the same layer composition, that is $m_{ij}^{\left[\alpha \right]}=m_{jk}^{\left[\alpha \right]}=1$ for all *α*, then *β*_*ik*,*jk*_ = 0 and $z^{\beta_{ik,jk}}=1$ indicating that we attribute no cost penalty to this configuration.

The term *σ*_*ij*\*k*_ includes contributions from paths of length one ($M_{ij}$) and two (${\hat{M}}_{ijr}$) between node *i* and node *j* that pass through node *r* with *r* ≠ *k*, i.e.

$$\begin{array}{c} \sigma_{ij\setminus k}=\frac{1}{\mu }\left[M_{ij}+\sum_{r\neq k}{\hat{M}}_{ijr}\right], \end{array}$$ (6)

where *μ* is a normalization constant with *μ* = max(1, *ν*) with

$$\begin{array}{c} \nu =\min(\sum_{r\neq k}A_{ir},\sum_{r\neq k}A_{jr}). \end{array}$$ (7)

Similarly to the modularity measure [33], term $M_{ij}$ evaluates the significance of the observed multilink ${\vec{m}}_{ij}$ against its expectation and, ${\hat{M}}_{ijr}$ evaluates the significance of two non-trivial multilinks ${\vec{m}}_{ir},{\vec{m}}_{jr}$ connecting respectively node *i* and node *j* to a common node *r* ≠ *k* against their expectations. These terms are

$$\begin{array}{c} M_{ij}=\left(A_{ij}-p_{ij}^{{\vec{m}}_{ij}}\right)z^{\beta_{ij,ij}}\delta \left(A_{ij},1\right) \end{array}$$ (8)

and

$$\begin{array}{c} {\hat{M}}_{ijr}=\left(A_{ir}A_{jr}-p_{ir}^{{\vec{m}}_{ir}}p_{jr}^{{\vec{m}}_{jr}}\right)z^{\beta_{ir,jr}}\delta \left(A_{ir}A_{jr},1\right), \end{array}$$ (9)

where *β*_*ij*,*rs*_ is given by Eq (5), and *δ*(*x*, *y*) is the Kronecker delta (i.e. *δ*(*x*, *y*) = 1 for *x* = *y* and *δ*(*x*, *y*) = 0 otherwise). The term $z^{\beta_{ij,rs}}$ puts a cost to the paths that are created using different layers. The expectation of multilink ${\vec{m}}_{rs}$ is given by the probability $p_{rs}^{{\vec{m}}_{rs}}$, which is evaluated using maximum entropy ensembles preserving the degree of node *i* and node *j* in each layer *α*, and the multilinks ${\vec{m}}_{ik},{\vec{m}}_{jk}$ (see S1 Appendix for further details).

Summing up, the parameter *ϵ* can be used to tune the contribution to the similarity *S*_*ij*,*jk*_ coming from the composition of the two incident multilinks and the contribution coming from the local clustering of the multiplex network in proximity of the wedge *ijk*. In particular the smaller is *ϵ* the larger is the contribution due to the local clustering of the multiplex network.

From numerical experimentation, we observed that the time to evaluate the similarity matrix *S*_*ik*,*jk*_ using our MATLAB code grows as *N*^3^ for multiplex networks with the same number of layers and increases linearly with the number of layers *M* making our method suitable for small to medium size networks (see S1 Fig).

### Multilink communities

From the *L* × *L* similarity matrix *S*_*ik*,*js*_, we construct a dendrogram via single linkage hierarchical clustering. The dendrogram contains information about the multiplex structure which cannot be obtained from the aggregated network. Finally the multilink communities are determined by cutting the dendrogram at a height that correspond to an optimal value of a appropriate score function.

To obtain the multilink communities we desire to use a score function that does not use any a priori assumptions about the multilink composition. To this end we have considered a score function used on single-layer link-community detection methods, i.e. the link modularity Q [26] (see S1 Appendix for its definition). An alternative choice could be to choose the partition density *D* used in [25]. The optimal partition is defined by the maximum value of Q obtained when considering all the heights in the dendrogram (see S4 Fig for typical profiles of this link modularity on real datasets).

Once every multilink is associated to a given multilink community we can assign to each node a *community activity* given by the number of communities to which its incident multilinks belong.

## Results and discussion

### A simple example

**Fig 1:**
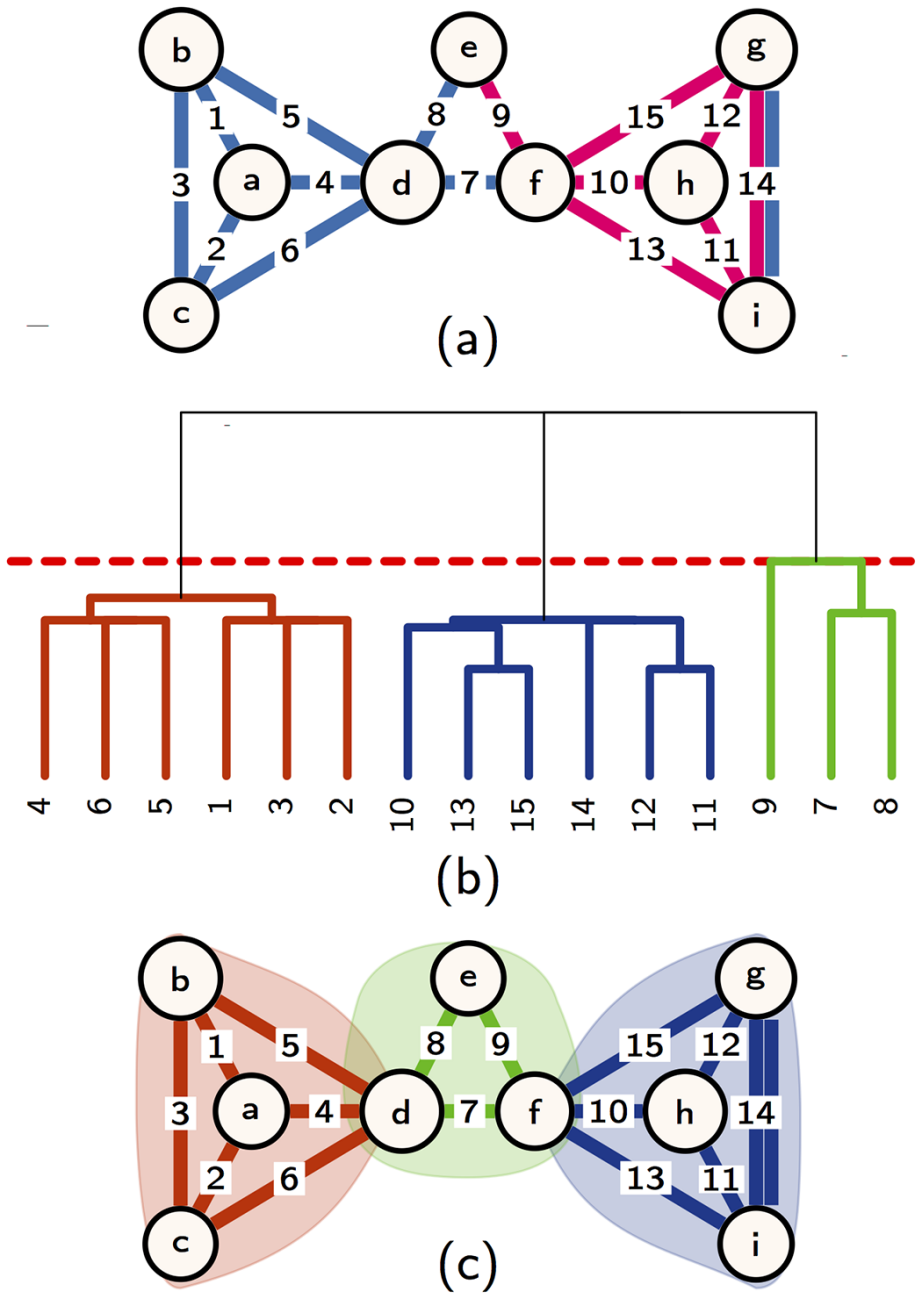
A two layer multiplex and its partition into multilink communities. (A) A simple two layer multiplex networks where the links in blue correspond to one layer and the dark pink ones to another layer. (B) Dendrogram of the multiplex network obtained from the multilink similarity. The dashed red line shows the maximum link modularity used to define the link communities. (C) Panel C shows the partition of the multiplex network into three communities revealing that communities can be formed by a single (community {*a*, *b*, *c*, *d*}) or multiple layers (community {*d*, *e*, *f*}) and that the nodes communities are independent on the node activity (node *d* belongs to two community and is active in one layer, node *g* is belongs to one community and is active in one layer). The colors of the dendrogram branches in panel B and of the color of the multilinks of the multiplex network in panel C indicate the different multilink communities. The multilink communities were detected using *ϵ* = 0.4, *z* = 0.6.

The community activity of a node resulting from the multilink community detection method is independent on its layer activity. To illustrate this property we consider the multilayer network shown in Fig 1A decomposed in three multilink communities Fig 1C) detected using the parameters *ϵ* = 0.4 and *z* = 0.6. Node *d* is active in a single layer but belongs to two multilink communities. On the contrary node *g* is active in two layers but belongs to just one community.

Additionally the communities can be formed by interactions existing only in one layer or in multiple layers. For instance the community formed by the nodes {*e*, *d*, *f*} of the multiplex network shown in Fig 1A, only exist due to the combination of different layers in the multiplex. On the contrary the community formed by the nodes {*a*, *b*, *c*, *d*} include only links of a single layer.

The dendrogram in Fig 1B shows the hierarchical structure of the link communities of the multiplex network in Fig 1A and reveals the multilayer nature of the network also in the case of this very symmetrical and clustered topology. In fact, the left and right communities of Fig 1C, although they play the same role in the aggregated network, have a different decomposition into multilink sub-communities. There are two factors that contribute to this difference. The right community has a multilink formed by two layers (multilink 14) which are no present in the other community. The second factor is more subtle and it would generate differences in the hierarchical structure even if the community on the right included only links existing in a single layer (see S1 Appendix and S2 Fig for details).

### Florentine families

**Fig 2:**
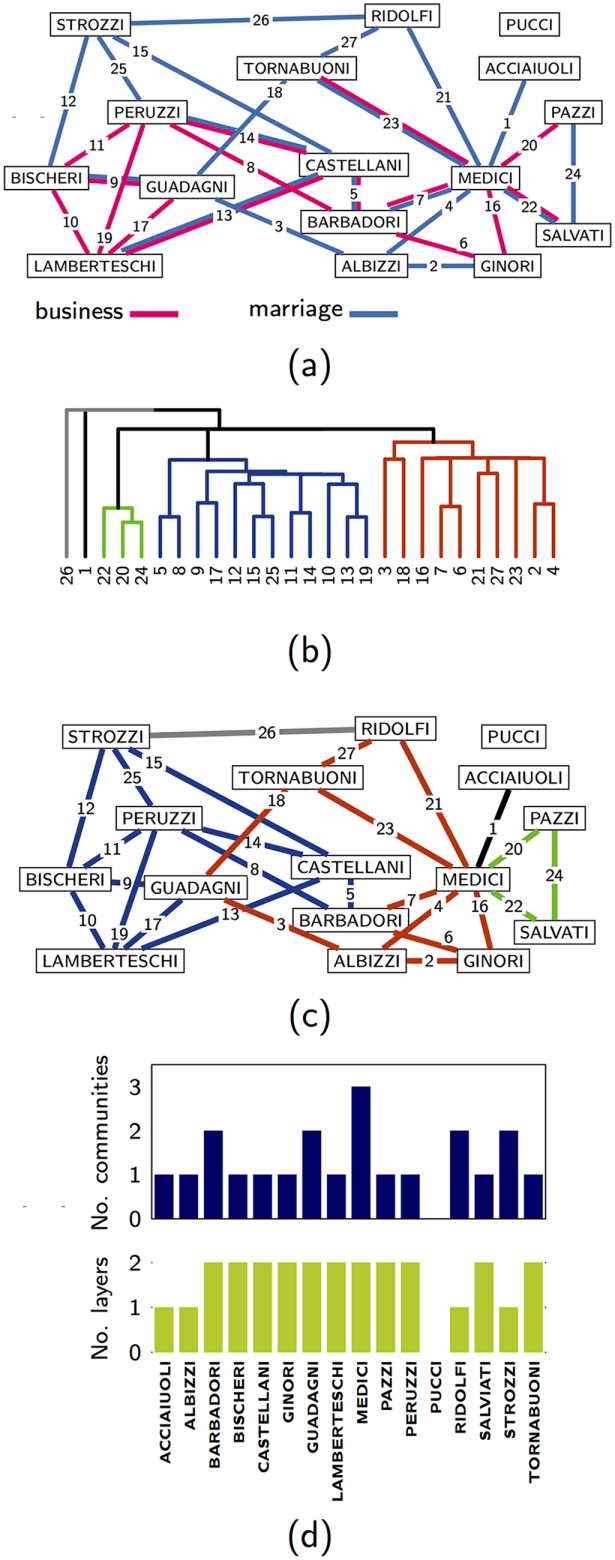
Multinlink communities for the florentine families. (A) The Florentine Families Multiplex Network describing the business and marriage alliances of the XV century florentine families. (B) Heat map displaying the multilink similarity matrix and its relative dendrogram. (C) Partition of the Florentine Families multiplex network into five multilink communities. (D) Layer and community activity of the different families. The Medici family is characterized by achieving the maximum of the community activity. The multilink communities are detected using *ϵ* = 0.4, *z* = 0.6.

The Florentine Families Multiplex Network [34] consist of *M* = 2 two layers, one layer describes the business dealings between *N* = 16 florentine families in the XV century, the other layer their alliances due to marriages. Fig 2A shows these relationships between the families. Fig 2B shows the dendrogram describing the multilink communities for *ϵ* = 0.5, *z* = 0.6 (see S3 Fig for the dependence of the number of clusters on *ϵ* and *z*).

The two detected single multilink communities correspond to two different scenarios (Fig 2C). The multilink between the Strozzi and the Ridolfi family establish an interaction between two families which have connections between different clusters; the multilink between the Acciaiuoli and the Medici family is a leaf of the multiplex network, being the only multilink connecting the Acciaiuoli family to the rest of the multiplex network.

For each family we compare their *layer activity* and their *community activity* (Fig 2D). We observe that families with high community activity are powerful brokers between different communities. Most relevantly, the Medici play a pivotal role as they are brokers between three different communities. The Barbadori and the Guadagni family have the same community activity as the Ridolfi and the Strozzi family but while the first two are connected in both layers the latter two are connected to the other families exclusively in one layer (the marriage alliances).

### Multiplex connectome of C. elegans

**Fig 3:**
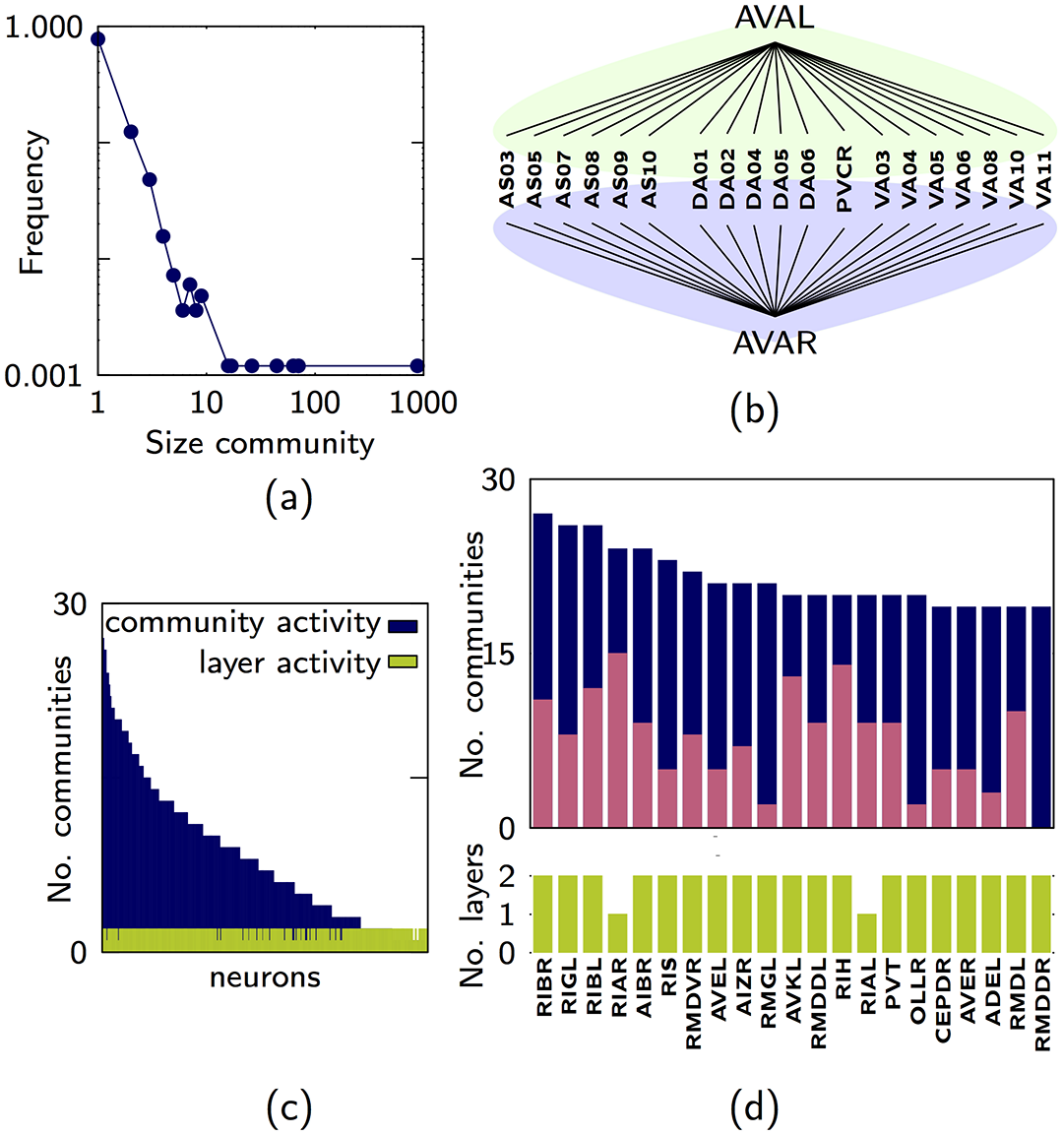
Properties of the multilink commuities of the *C. elegans*. (A) Distribution of the communities sizes for the Multiplex Connectome of *C. elegans*. (B) The two most similar sub-communities contained in the largest multilink community. (C) Neurons ranked in decreasing order of their community activity. (D) Layer and community activity for the top ranked neurons. (the contribution of communities with single multilinks is in pink while the contributions of communities with more than one multilink is in blue). The multilink communities are detected using *ϵ* = 0.4, *z* = 0.6.

The Multiplex Connectome of *C. elegans* [35, 36] has two layers *M* = 2, the chemical synapses and the gap junctions describing the interactions between *N* = 279 neurons. As an example, we obtained the multilink communities for *ϵ* = 0.4 and *z* = 0.6. The multiplex has 845 multilink communities of which 652 (about 77%) are made of single multilinks. The distribution of the sizes of the communities is broad. (Fig 3A). The largest community is formed by 878 multilinks followed communities including 67 links and 51 links. Although there is a large dominant community in the multiplex network, the internal structure of this community can be investigated via the dendrogram. We noticed that the ADAL and ADAR are the neurons that cluster first with some of their neighboring neurons (Fig 3B) for all values of *z*.

This multiplex network has neurons which have large community activity (Fig 3C). By ranking the neurons according to their community activity we find in the first two positions the RIBR and RIBL neurons, which are head interneurons connected via gap junctions to multiple other neuron classes, suggesting that these neurons play a role in brokering between different communities (Fig 3D).

### European multiplex air transport network

The European Multiplex Air Transport Network [14] comprises of *N* = 417 European airports and *M* = 37 layers corresponding to the airlines that have flight connections between these airports. The total number of multilink describing these connections is 2953. For the case that *ϵ* = 0.4 and *z* = 0.6, our algorithm obtains 1790 multilink communities. The largest community includes 723 nodes, about 24% of the total number of multilinks. The smallest communities are made of single multilinks and there are 1696 of them, about 57% of the multilinks.

**Fig 4:**
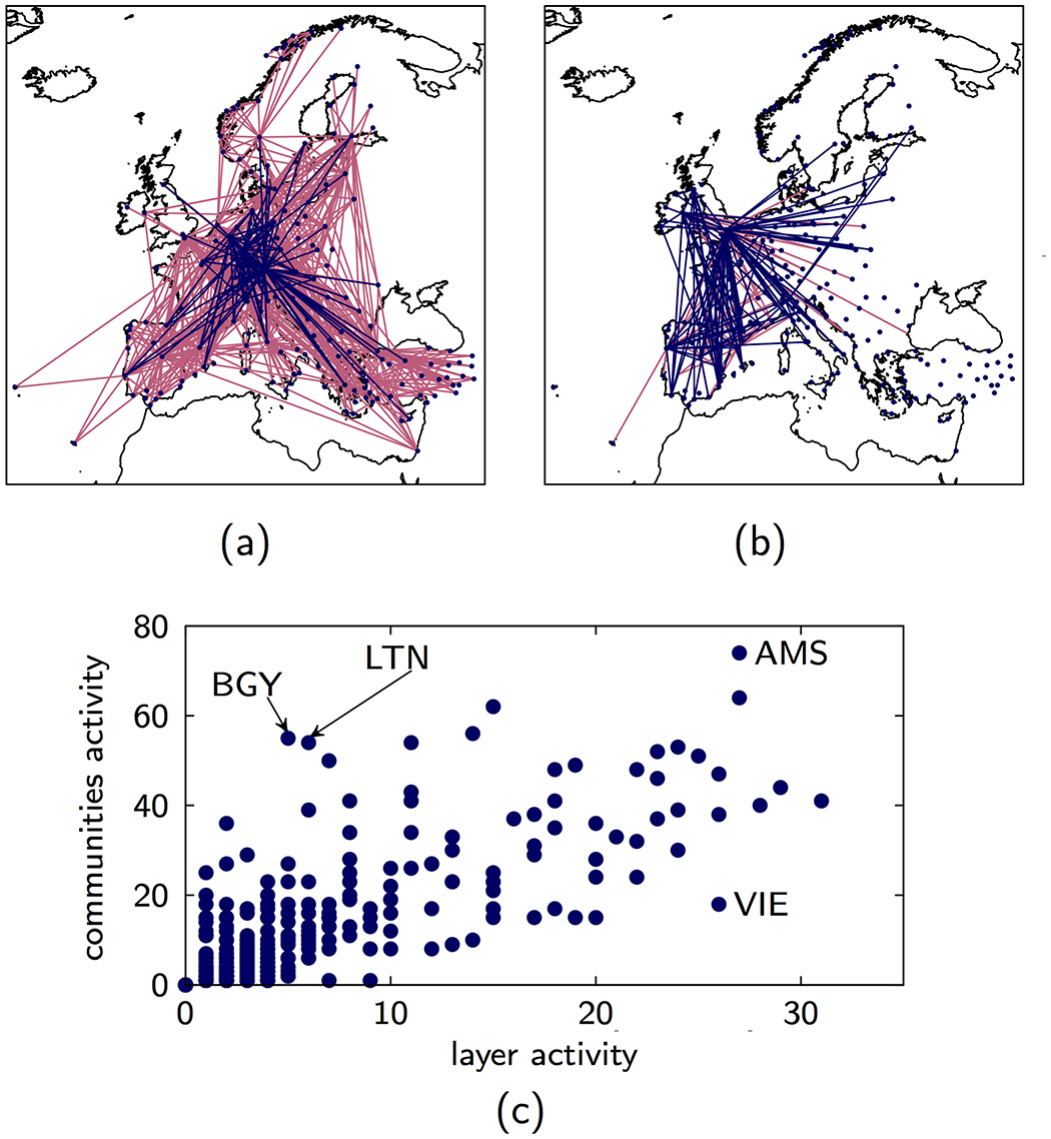
Community activity vs layer activity for the EU Airports multiplex. (A) Largest link community of the European Multiplex Air Transportation Network. Lufthansa’s flights are shown in blue, the other airlines in pink. (B) Second largest link community. Ryanair’s flights are shown in blue, the other airlines in pink. (C) Community vs. layer activity of the EU airports. While the layer activity appears to have a positive correlation with the communities activity, there are large differences in the communities activity between airports with large layer activity (compare for instance Amsterdam (AMS) and Vienna (VIE)). The multilink communities are detected using *ϵ* = 0.4, *z* = 0.6.

We observe that the main communities have very different composition in term of single layers. Fig 4A and 4B shows the two largest communities. All the airlines (layers) contribute to the structure of the largest community (Fig 4A). The second largest community has a very different structure, only few airlines contribute to this community.

When comparing the airports and their community activity, we observe (Fig 4C) that while large layer activity, an airport serving multiple airline companies, seems to be correlated to high community activity, there is a significant variability in the community of airports that are active in many layers. For example Vienna (VIE) and Amsterdam (AMS) have a comparable layer activity but very different community activity. Similarly there are airports with small layer activity but significant community activity, for example Luton (LTN) and Bergamo (BGY) airports. This indicates that the airports might adopt different strategies to broker between different communities. These strategies might involve serving flights of many airline companies or serving flights of relatively fewer airline companies.

The parameter *ϵ* and *z* can be tuned to change the number of multilinks communities with larger values of *z* penalizing communities where multilinks have different composition $\vec{m}$, while larger values of *ϵ* allows to weight more the contribution to the multilink similarity coming from the local neighborhood of the wedge *ikj*. The dependence of the number of communities with the parameters *z* and *ϵ* for the three data sets is investigated in the Supporting Information (S1 Appendix). Our choice of the parameters *ϵ* = 0.4 and *z* = 0.6 is dictated by the desire of having multilink communities that might span several layers (excluding very large values of *z*) extracted from a similarity matrix significantly affected by the local neighborood of the wedges (excluding very low values of *ϵ*). Additionally these parameter values for the analysed real datasets lead to score function profiles with a well defined maximum (see S3 and S4 Figs).

However we point out that *ϵ* and *z* remain tunable parameters that can be chosen accordingly to the dataset and the scientific problem under study as it is done for instance with the resolution parameter in modularity optimization algorithms.

### Composition of the multilink communities

To investigate whether the communities are formed exclusively by links of a single layer or include links of several layers we introduce the *layer specificity* $x_{c}^{\left[\alpha \right]}$ which is the fraction of multilinks in a community *c* which include a link in layer *α*. Therefore $x_{c}^{\left[\alpha \right]}=1$ indicates that all the multilinks of a community include a link in layer *α*, while $x_{c}^{\left[\alpha \right]}=0$ indicates that the community does not include any link in layer *α*. Note that since a single multilink can include links of different layers, the sum of the layer specificity $x_{c}^{\left[\alpha \right]}$ for community *c* in general do not add to one.

**Fig 5:**
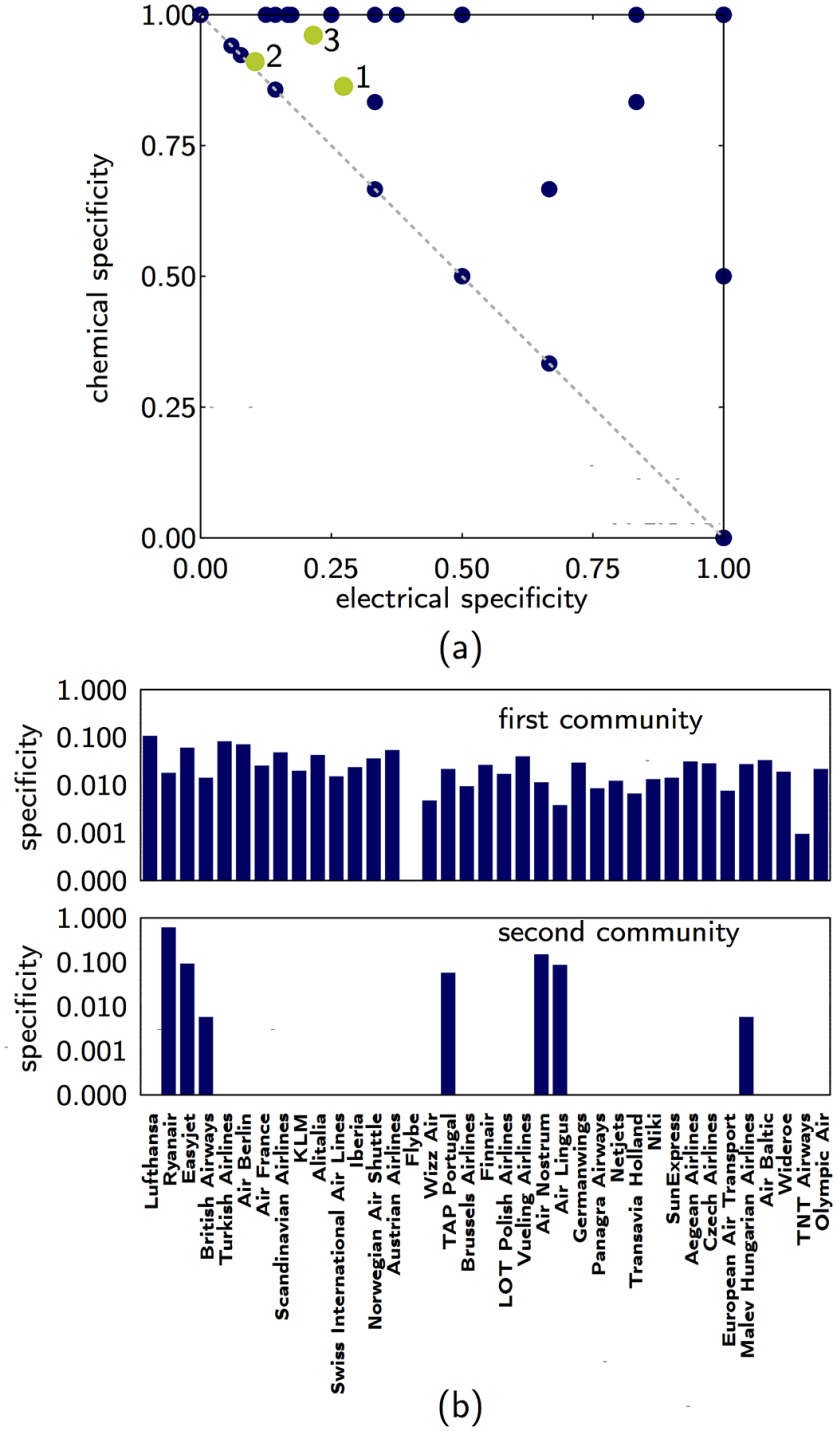
Specificity of the *C. elegans* and the EU Airlines. (A) Specificity for the communities in the Multiplex Connectome of *C. elegans*. (B) Specificity for the first and second largest communities for the European Air Transportation Network. Both panels indicate a large variability in the layer composition of different communities. The multilink communities are detected using *ϵ* = 0.4, *z* = 0.6.

In the Multiplex Connectome of *C. elegans* we observe that many communities are exclusively formed by one type of multilink, however, the three largest communities have a multiplex nature as they include different types of multilinks (see Fig 5A where the larger communities are indicated by the labels 1, 2, 3 in order of decreasing size).

In the European Multiplex Air Transportation Network, the largest community, apart from Flybe, contains flights from all other airlines (Fig 5B). The largest contribution comes from Lufthansa with an specificity of 0.10 followed by Turkish Airlines with 0.07 specificity. The second largest community has a different structure, in this case only seven airlines contribute to the community, the largest contribution is from Ryanair with a specificity of 0.60. In this multiplex, low-cost airlines like Ryanair, Easyjet and Wideroe have high specificity (often equal to 1) in many communities. However these airlines rarely have high specificity in the same community. This is a consequence of the competition between low-cost airline companies as they tend to differentiate each other by having unique flights to some destinations.

## Conclusion

Our method reveal the richness of multiplex networks at their mesoscale structure. This is achieved by associating to each pair of incident multilinks a similarity measure based on the comparison of the local connectivity of two multilinks against a null model. Our intrinsically multiplex community detection method allow us to associate to each node multiple communities independently on its layer activity. Specifically we can have nodes active exclusively in one layer and belonging to multiple communities or active in many layers but belonging only to few communities. The proposed method is here applied to several real datasets revealing that the mesoscale structure of a multiplex can be organized via communities containing links in many different layers and, at the same time, communities having one predominant layer. This suggests that the mesoscale organization of multiplex networks has a rich mesoscale structure that is not captured by methods that aim at compressing the information on few single layers.

## Supporting information

S1 Appendix Description of the multilink community method. Detailed description of the Multilink Community detection algorithm and additional results obtained by applying the Multilink Community detection algorithm to benchmark multiplex networks models and real multiplex networks datasets.
(PDF)

S1 Fig Computational complexity as a function of the number of nodes and number of layers. (A) Average CPU time elapsed to evaluate all the elements of the matrix *S*_*ik*,*jk*_ as a function of the number of nodes for two families of multiplexes. Bottom line the multiplexes are defined by 〈*k*^[*α*]^〉 = 3 (bottom line) and 〈*k*^[*α*]^〉 = 3, the dashed lines show the best fit via *f*(*N*) = *a* + *bN*^*γ*^. (B) Average CPU time as a function of *M*. The horizontal lines correspond to multiplexes with, from bottom to top, *N* = 100, 500, 1000, 1500, 2000, 2500.
(PDF)

S2 Fig Example of a two layer multilink community. (A) A simple two layer multiplex network (purple and ochre links) and its multilink communities (shaded areas) and (B) its dendrogram obtained from the multilink similarity. The dashed red line shows the maximum link modularity used to define the link communities.
(PDF)

S3 Fig Variation of the cluster size against the *z* and *ϵ* parameters. Variation of the number of clusters as a function of *z* with given *ϵ* (right panels) and as a function of *ϵ* with given *z* (left panels). The top panels show the Florentine families, the middle panels the *C. elegans* and the bottom panels the EU-airports multiplexes. In the left panels data are shown for *ϵ* = 0.4 (blue triangles), *ϵ* = 0.5 (pink circles) and *ϵ* = 0.6 (green squares). In the right panels data are shown for *z* = 0.4 (blue triangles), *z* = 0.5 (pink circles) and *z* = 0.6 (green squares). The red circles show the values used in the main manuscript.
(PDF)

S4 FigNumber of clusters against the score function Q (link–modularity).(A) Florentine Families Multiplex Network (*ϵ* = 0.5, *z* = 0.6). (B) The Multiplex Connectome of *C. elegans* (*ϵ* = 0.4, *z* = 0.6) and (C) for the European Multiplex Air Transportation Network (*ϵ* = 0.4, *z* = 0.6). The maximum of Q determines the number of clusters which define the multlink communities of the multiplex network.(PDF)

S5 Fig Aggregated degree vs. community activity. (A) Multiplex Connectome of *C. elegans* and (B) for the European Mutliplex Air Transportation Network.
(PDF)

S1 Data Florentine family multiplex dataset. (ZIP)

S2 Data EU air transportation multiplex dataset. (ZIP)
