# Supplementary material for: Multilink communities of multiplex networks
Source: PLoS One. 2018 Mar 20;13(3):e0193821. doi: 10.1371/journal.pone.0193821 (PMC5860749; doi:10.1371/journal.pone.0193821)
Supplement: S1 Appendix — Detailed description of the Multilink Community detection algorithm and additional results obtained by applying the Multilink Community detection algorithm to benchmark multiplex networks models and real multiplex networks datasets. (PDF) [file pone.0193821.s001.pdf]

S1 Appendix Supplementary Information on "Multilink Communities of Multiplex Networks"

Raúl J Mondragón[1], Jacopo Iacovacci[2] and Ginestra Bianconi[2]

March 10, 2018

[1] School of Electronic Engineering and Computer Science, Queen Mary University of London, London, United Kingdom

[2] School of Mathematical Sciences, Queen Mary University of London, London, United Kingdom

In this Supplementary Information we give further details on the Multilink Community detection algorithm proposed in the main body of the paper. Moreover we provide additional results obtained by applying the Multilink Community detection algorithm to benchmark multiplex networks models and real multiplex networks datasets. In this way we cover the dependence of the algorithm on the parameters ϵ and z and the algorithmic complexity of the algorithm. The codes implementing the Multilink Community detection method are freely available at the website `https://github.com/ginestrab`. All the datasets are freely available at `http://deim.urv.cat/~manlio.dedomenico/data.php`.

1 Detailed description of the Multilink Community detection algorithm

1.1 General considerations

The similarity between any two incident multilinks of the multiplex network is the basic element of the multilink community detection algorithm. The similarity matrix is used to perform a hierarchical clustering of the multilinks, ultimately finding the multilink communities as described in the main body of the paper. In the same spirit as in Ref. [1] the similarity matrix is constructed by comparing the local neighborhood of each pair of incident multilinks to a maximum entropy null model for the multiplex network.

In this section we give further information on the maximum entropy null model that we used to evaluate the similarity between any two incident multilinks. This model extends previous results on exponential random graphs of single [2] and multiplex networks [3, 4].

1.2 Multiplex network

Let us consider a multiplex network $\vec{G} = (G^{[1]}, G^{[2]}, \ldots, G^{[\alpha]}, \ldots, G^{[M]})$ formed by N nodes and M layers $\alpha = 1, 2, \ldots, M$. . Every layer α is formed by a undirected network with adjacency matrix $\mathbf{a}^{[\alpha]}$. Every pair of nodes (i, j) is connected by a multilink [3, 4]

$$\vec{m}_{ij} = \left(m_{ij}^{[1]}, m_{ij}^{[2]}, \ldots, m_{ij}^{[\alpha]} \ldots m_{ij}^{[M]}\right), \tag{S-1}$$

with $m_{ij}^{[\alpha]} = a_{ij}^{[\alpha]}$ indicating in which layers of the multiplex network the two nodes are connected. Whenever node i and node j are connected at least in one layer, i.e. $\vec{m} \neq \vec{0}$, we say that they are connected by a non-trivial multilink.

The aggregated network $\hat{G}$ is the single network in which any two nodes are connected if they are linked at least in one layer of the multiplex network. The adjacency matrix $\mathbf{A}$ of the aggregated network $\hat{G}$ has elements

$$A_{ij} = \theta\left(\sum_{\alpha=1}^{M} a_{ij}^{[\alpha]}\right), \tag{S-2}$$

where $\theta(x)$ is the step function $\theta(x) = 1$ if $x > 0$ and $\theta(x) = 0$ if $x \leq 0$. We indicate with $L = \sum_{i<j} A_{ij}$ the total number of links of the aggregated network, or equivalently the number of non-trivial multilinks.

1.3 Multilink similarity

In order to detect the multilink communities we assign a non zero similarity $S_{ik,jk}$ to every pair of incident multilinks connecting respectively the generic nodes i and k and j and k. The non-zero similarities $S_{ik,jk}$ are given by

$$S_{ik,jk} = \epsilon\sigma_{ijk} + (1-\epsilon)\sigma_{ij\backslash k}. \tag{S-3}$$

where σ_{ijk} evaluates the contribution of the two incident multilinks while $\sigma_{ij\backslash k}$, evaluates instead the contribution due to the existence of other multilinks, joining node i and node j directly or by paths of length two excluding node k. The parameter $\epsilon \in (0,1)$ tunes the relative importance between these two contributions. The term σ_{ijk} is expressed as

$$\sigma_{ijk} \quad = \quad z^{\beta_{ik,jk}}, \tag{S-4}$$

with

$$\beta_{ij,rs} = 1 - \frac{\sum_{\alpha=1}^{M} m_{ij}^{[\alpha]} m_{rs}^{[\alpha]}}{M}. \tag{S-5}$$

The term $\sigma_{ij\backslash k}$ includes contributions from paths of length one ($\mathcal{M}_{ij}$) and two ($\hat{\mathcal{M}}_{ijr}$) between node i and node j that pass through node r with $r \neq k$, i.e.

$$\sigma_{ij\backslash k} \quad = \quad \frac{1}{\mu}\left[\mathcal{M}_{ij} + \sum_{r\neq k} \hat{\mathcal{M}}_{ijr}\right], \tag{S-6}$$

where μ is a normalization constant with $\mu = \max(1, \nu)$ with

$$\nu = \min\left(\sum_{r\neq k} A_{ir}, \sum_{r\neq k} A_{jr}\right). \tag{S-7}$$

Similarly to the modularity measure [1], term $\mathcal{M}_{ij}$ evaluates the significance of the observed multilink $\vec{m}_{ij}$ against its expectation and, $\hat{\mathcal{M}}_{ijr}$ evaluates the significance of two non-trivial

multilinks $\vec{m}_{ir}, \vec{m}_{jr}$ connecting respectively node i and node j to a common node $r \neq k$ against their expectations. These terms are

$$\begin{aligned} \mathcal{M}_{ij} &= (A_{ij} - p_{ij}^{\vec{m}_{ij}}) z^{\beta_{ij,ij}} \delta(A_{ij}, 1), \\ \hat{\mathcal{M}}_{ijr} &= (A_{ir}A_{jr} - p_{ir}^{\vec{m}_{ir}} p_{jr}^{\vec{m}_{jr}}) z^{\beta_{ir,jr}} \delta(A_{ir}A_{jr}, 1), \end{aligned} \tag{S-8}$$

where $\beta_{ij,rs}$ is given by Eq. $(S-5)$, and $\delta(x, y)$ is the Kronecker delta (i.e. $\delta(x, y) = 1$ for $x = y$ and $\delta(x, y) = 0$ otherwise). The expectation of multilink $\vec{m}_{rs}$ is given by the probability $p_{rs}^{\vec{m}_{rs}}$, which is evaluated using maximum entropy ensembles.

The null model should not change the multilinks $\vec{m}_{ik}$ and $\vec{m}_{jk}$ determining the connection of nodes i and j with node k. This restriction fixes the connections between node i and k and node j and k but it does not restrict the connections between nodes i and j and their other neighbors. To capture the local structure on layer α, the null model should preserve the number of neighbors of nodes i and j in each layer α, that is their degree $q_i^{[\alpha]}$ and $q_j^{[\alpha]}$, however, except from node k, the neighbors are selected at random from the remaining $N - 2$ nodes. Therefore the maximum entropy model is preserving the degree of node i and node j in each layer α, and the multilinks $\vec{m}_{ik}, \vec{m}_{jk}$.

1.4 Maximum entropy ensemble

The considered maximum entropy ensemble is characterised by the probability $P(\vec{G})$ assigned to each possible multiplex network $\vec{G}$ determined by the set of adjacency matrices $\tilde{\mathbf{a}}^{[\alpha]}$ with $\alpha = 1, 2, \ldots, M$. This probability is found by maximising the entropy S which is the logarithm of the number of typical multiplex networks in the ensemble,

$$S = -\sum_{\vec{G}} P(\vec{G}) \ln P(\vec{G}) \tag{S-9}$$

given the set of structural constraints under consideration. These constraints are

$$\begin{aligned} \sum_{\vec{G}} \left(P(\vec{G}) \sum_{r \neq k} \tilde{a}_{ir}^{[\alpha]} \right) &= q_i^{[\alpha]} - a_{ik}^{[\alpha]}, \\ \sum_{\vec{G}} \left(P(\vec{G}) \sum_{r \neq k} \tilde{a}_{jr}^{[\alpha]} \right) &= q_j^{[\alpha]} - a_{jk}^{[\alpha]}, \end{aligned} \tag{S-10}$$

with $\alpha = 1, 2, \ldots, M$. By introducing the Lagrangian multipliers $\lambda_i^{[\alpha]}, \lambda_j^{[\alpha]}$ with $\alpha = 1, 2, \ldots, M$ the probability $P(\vec{G})$ can be written as

$$P(\vec{G}) = \frac{1}{Z} e^{-\sum_{\alpha=1}^{M} H_{ij}^{[\alpha]}}, \tag{S-11}$$

where the partition function Z is a normalization constant, and $F_{ij}^{[\alpha]}$ is given by

$$\begin{aligned} H_{ij}^{[\alpha]} &= \lambda_i^{[\alpha]} \left(\sum_{r \neq \{k,i,j\}} \tilde{a}_{ir}^{[\alpha]} \right) + \lambda_j^{[\alpha]} \left(\sum_{r \neq \{k,i,j\}} \tilde{a}_{jr}^{[\alpha]} \right) \\ &\quad + (\lambda_i^{[\alpha]} + \lambda_j^{[\alpha]}) \tilde{a}_{ij}^{[\alpha]}. \end{aligned} \tag{S-12}$$

The marginal probability of single links of nodes i and node j in each layer α are given, for $r \neq \{i, j, k\}$ by

$$\begin{aligned} p_{ir}^{[\alpha]} &= \sum_{\vec{G}} \left(P(\vec{G}) \tilde{a}_{ir} \right) = \frac{e^{-\lambda_i^{[\alpha]}}}{1 + e^{-\lambda_i^{[\alpha]}}}, \\ p_{jr}^{[\alpha]} &= \sum_{\vec{G}} \left(P(\vec{G}) \tilde{a}_{jr} \right) = \frac{e^{-\lambda_j^{[\alpha]}}}{1 + e^{-\lambda_j^{[\alpha]}}}, \end{aligned} \tag{S-13}$$

and by

$$p_{ij}^{[\alpha]} = \sum_{\vec{G}} \left(P(\vec{G}) \tilde{a}_{ij} \right) = \frac{e^{-\lambda_i^{[\alpha]} - \lambda_j^{[\alpha]}}}{1 + e^{-\lambda_i^{[\alpha]} - \lambda_j^{[\alpha]}}}. \tag{S-14}$$

The Lagrangian multipliers $\lambda_i^{[\alpha]}$ and $\lambda_j^{[\alpha]}$ are determined by the constraints in Eq. (S-10) that, in terms of the marginals is

$$\begin{aligned} \left(\sum_{r \neq \{j,k\}} p_{ir}^{[\alpha]} \right) + p_{ij}^{[\alpha]} = q_i^{[\alpha]} - a_{ik}^{[\alpha]}, \\ \left(\sum_{r \neq \{i,k\}} p_{jr}^{[\alpha]} \right) + p_{ij}^{[\alpha]} = q_j^{[\alpha]} - a_{jk}^{[\alpha]}. \end{aligned} \tag{S-15}$$

Finally this maximum entropy ensemble allow us to determine the probability $p_{ir}^{\vec{m}_{ir}}$ and $p_{jr}^{\vec{m}_{jr}}$ of the multilinks $\vec{m}_{ir}, \vec{m}_{jr}$ which are given respectively by

$$\begin{aligned} p_{ir}^{\vec{m}_{ir}} &= \sum_{\vec{G}} P(\vec{G}) \prod_{\alpha=1}^{M} \left(\tilde{a}_{ir}^{[\alpha]} m_{ir}^{[\alpha]} + (1 - \tilde{a}_{ir}^{[\alpha]})(1 - p_{ir}^{[\alpha]}) \right) \\ &= \prod_{\alpha=1}^{M} \left(p_{ir}^{[\alpha]} m_{ir}^{[\alpha]} + (1 - m_{ir}^{[\alpha]})(1 - p_{ir}^{[\alpha]}) \right), \end{aligned} \tag{S-16}$$

and

$$\begin{aligned} p_{jr}^{\vec{m}_{jr}} &= \sum_{\vec{G}} P(\vec{G}) \prod_{\alpha=1}^{M} \left(\tilde{a}_{jr}^{[\alpha]} m_{jr}^{[\alpha]} + (1 - \tilde{a}_{jr}^{[\alpha]})(1 - p_{jr}^{[\alpha]}) \right) \\ &= \prod_{\alpha=1}^{M} \left(p_{jr}^{[\alpha]} m_{jr}^{[\alpha]} + (1 - m_{jr}^{[\alpha]})(1 - p_{jr}^{[\alpha]}) \right). \end{aligned} \tag{S-17}$$

1.5 Multilink communities

From the $L \times L$ similarity matrix $S_{ik,js}$, we construct a dendrogram via single linkage hierarchical clustering. The multilink communities are obtained by cutting the dendrogram at a height that correspond to the maximum value of the link modularity $\mathcal{Q}$.

The link modularity $\mathcal{Q}$ [5] is given by

$$\mathcal{Q} = \frac{1}{\sum_\ell d_\ell} \sum_{\ell,\ell'} \left[W_{\ell,\ell'} - \frac{d_\ell d_{\ell'}}{\sum_\ell d_\ell} \right] \delta(c_\ell, c_{\ell'}), \tag{S-18}$$

where $\mathbf{W}$ is the adjacency matrix of the line graph of the aggregated network and has elements $W_{\ell,\ell'} = 1$ if the link ℓ is incident to the link ℓ' while otherwise $W_{\ell,\ell'} = 0$. Additionally in Eq. (S-18) we indicate with d_ℓ the link-degree $d_\ell = \sum_{\ell'} W_{\ell,\ell'}$ and and with c_ℓ the cluster membership of the multilink corresponding to the link ℓ of the aggregated network. Finally $\delta(a,b) = 1$ if and only if $a = b$ otherwise $\delta(a,b) = 0$.

Once every multilink is associated to a given multilink community, each node is attributed a *community activity* given by the number of different communities to which its incident multilinks belong.

2 Application of the algorithm to benchmark models and algorithmic complexity

2.1 Computational Complexity

To estimate the computational complexity of our method we measured how long it takes (CPU time) to compute all the elements of the similarity matrix $S_{ik,jk}$ as a function of the number nodes N and the number of layers M. To measure the N dependance we constructed different multiplexes as follows. The layers of a multiplex are generated using a general power law growth model where all the layers follow the same power law with exponent -2.2. The networks were generated using the simples_fitness_pl function from the package igraph running under R (http://igraph.org/r/). In each individual layer, the label nodes were randomised to reduce any layer-layer correlation between the nodes. The number of links for the individual networks were fixed by considering the same average degree per layer, that is $\langle k^{[\alpha]}\rangle = k$.

Fig S-1A shows the time in seconds to evaluate all the elements of the similarity matrix for the case that $M = 3$ and $\langle k^{[\alpha]}\rangle = 3$ (bottom line) and $\langle k^{[\alpha]}\rangle = 4$ (top line). The dashed lines show the fit of the function $f(N) = a + bN^\gamma$ to the data points. The fit gave $\gamma = 3 \pm 0.12$ showing that the computational complexity increase as the cube of the number of nodes.

To show the dependance of the computational complexity with M, we created several multiplexes where all layers in the multiplex are identical so there is no variability in the average degree $\langle k^{[\alpha]}\rangle$ and in the aggregated degree $\langle K\rangle$. Fig S-1B shows the time against $M = 10, 20, 30, 40$. The figure shows six sets of multiplexes (horizontal lines) each corresponding to multiplexes with different number of nodes $N = 100, 500, 1000, 1500, 2000, 2500$. Each point in the figure shows the average time for 8 multiplexes generated with the same parameters (M, N, $\langle k^{[\alpha]}\rangle$, $\langle K\rangle$) and their standard deviation. From the numerical measurements it is clear that the CPU time increases linearly with M within the errors bars.

2.2 More on the benchmark multiplex network

In this paragraph we give some more background information on the multilink communities found for the simple multiplex network shown in Fig S-2A-B. In this multiplex network there is a difference between the structure of the right and left multilink communities shown in Fig S-2C, this difference is clearly seen in the dendrogram shown in Fig 1B. To explain this difference here we consider a a different multiplex network shown in Fig S-2A together and its partition into multilink communities (shaded areas). Although the community structure of this multiplex network is identical to Fig S-2C, its dendrogram (Fig S-2B) is again, not symmetric under the permutation of the right and left communities. The difference is due to the multiplexity of the network. In fact node f and node d play slightly different roles in their communities. Node f is

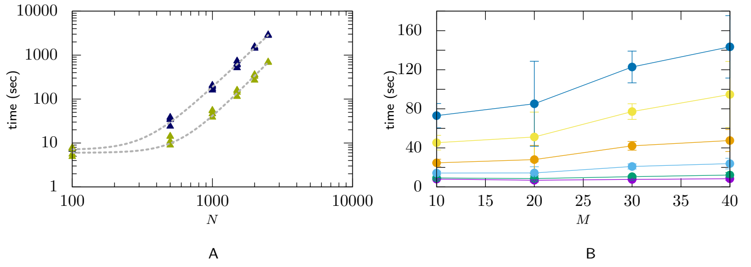

Figure S-1: **Computational complexity as a function of the number of nodes and number of layers.** (A) Average CPU time elapsed to evaluate all the elements of the matrix $S_{ik,jk}$ as a function of the number of nodes for two families of multiplexes. Bottom line the multiplexes are defined by $\langle k^{[\alpha]}\rangle = 3$ (bottom line) and $\langle k^{[\alpha]}\rangle = 3$, the dashed lines show the best fit via $f(N) = a + bN^{\gamma}$. (B) Average CPU time as a function of M. The horizontal lines correspond to multiplexes with, from bottom to top, $N = 100, 500, 1000, 1500, 2000, 2500$.

active in two different layers, while node d is active only in one layer. Our method distinguishes these two cases.

3 Application of the algorithm to real dataset and dependence on the parameters z and ϵ

3.1 General considerations

In this section we provide additional results regarding the application of the Multilink Communities detection Algorithm to the the three real datasets considered in the main text of the paper: the Florentine Families Multiplex Network, the Multiplex Connectome and the European Multiplex Air Transportation Network.

3.2 The number of multilink communities as a function of the parameters ϵ and z

The values of the parameters z and ϵ can be tuned to change the number and properties of the multilink communities. A very large value of z imposes a very strong penalty for multilink communities that have a very diverse composition of multilinks favouring for instance communities present only in one layer or communities overlapping in different layers. A very small value of ϵ reduces the contribution to the similarity element $S_{ik,jk}$ coming from the neighbourhood of the wedge ikj (path of distance two and one between nodes i and j not passing through node k).

In Fig S-3 we display the dependence of the number of communities with the parameter values for the three considered real multiplex networks. The parameters $\epsilon = 0.4$ and $z = 0.6$ chosen in our main text constitute a trade-off between having a value of z that is not too high allowing also multilayer communities to span across different layers and a value of ϵ that is not to small, emphasising the contribution of the local neighbourhood of the wedge ikj in determining the similarity $S_{ik,jk}$. However the parameters used in the manuscript were chosen for demonstration purposes and we expect that, when using the method introduced here, the most appropriate choice of the parameters z and ϵ will depend of the multiplex network under study. From our

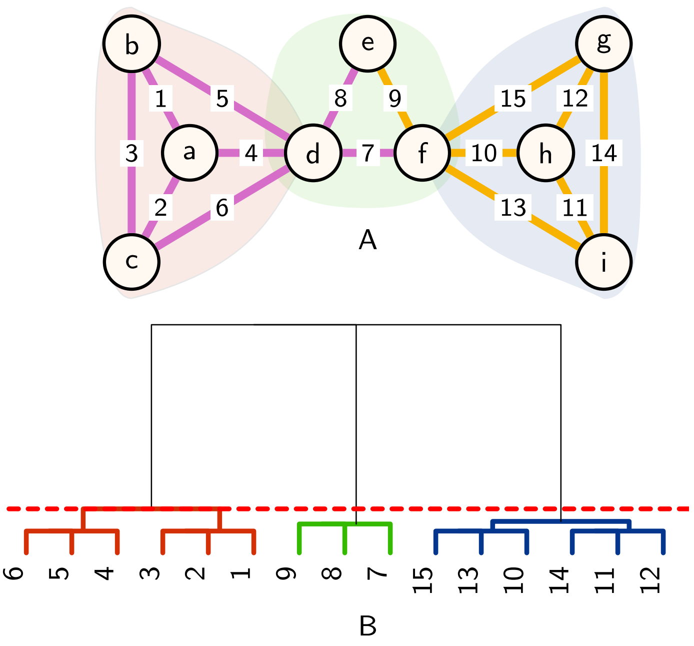

Figure S-2: **Example of a two layer multilink community.** (A) A simple two layer multiplex network (purple and ochre links) and its multilink communities (shaded areas) and (B) its dendrogram obtained from the multilink similarity. The dashed red line shows the maximum link modularity used to define the link communities.

numerical studies shown in Fig S-3 (left panel), we noticed that for many different values of the parameter z the method consistently divided the Florentine and EU-airports multiplexes into a fixed number of communities and that their is more variability in number of communities as the parameter ϵ varies (right panel).

We note here also that the parameters of $z = 0.6$ and $\epsilon = 0.4$ that we used in the manuscript correspond also to a relative stable number of communities as a function of the z parameter.

3.3 The score function profile of the analysed datasets

Once we calculate the similarity matrix $S_{ik,jk}$ and we construct its corresponding dendrogram, the multilink communities are determined by cutting the dendrogram at a height that corresponds to the maximum value of the score function $\mathcal{Q}$. In the datasets considered here, we observed that the profile of the link modularity $\mathcal{Q}$ (Fig S-4) displays a well defined global maximum, suggesting that the determination of the optimal partition is not questionable.

3.4 Aggregated degree vs. community activity

From the multilink communities we obtain the community activity of the nodes indicating for each node the number of multilink communities to which it belongs. A relevant question is whether the community activity of a node is correlated to its degree in the aggregated network $\hat{G}$. To this end in Figure S-5 we show the community activity versus the degree of the aggregated network for the Multiplex Connectome of *C. elegans* (Fig S-5A) and the European Multiplex Air Transportation Network (Fig S-5B). For small degrees, there is a significant positive correlation between these two quantities, but as the degree increases, the correlation diminishes.

References

[1] Mark EJ Newman. Modularity and community structure in networks. *Proceedings of the national academy of sciences*, 103(23):8577–8582, 2006.

[2] Juyong Park and Mark EJ Newman. Statistical mechanics of networks. *Physical Review E*, 70(6):066117, 2004.

[3] Ginestra Bianconi. Statistical mechanics of multiplex networks: Entropy and overlap. *Physical Review E*, 87(6):062806, 2013.

[4] Giulia Menichetti, Daniel Remondini, Pietro Panzarasa, Raúl J Mondragón, and Ginestra Bianconi. Weighted multiplex networks. *PloS one*, 9(6):e97857, 2014.

[5] TS Evans and R Lambiotte. Line graphs, link partitions, and overlapping communities. *Physical Review E*, 80(1):016105, 2009.

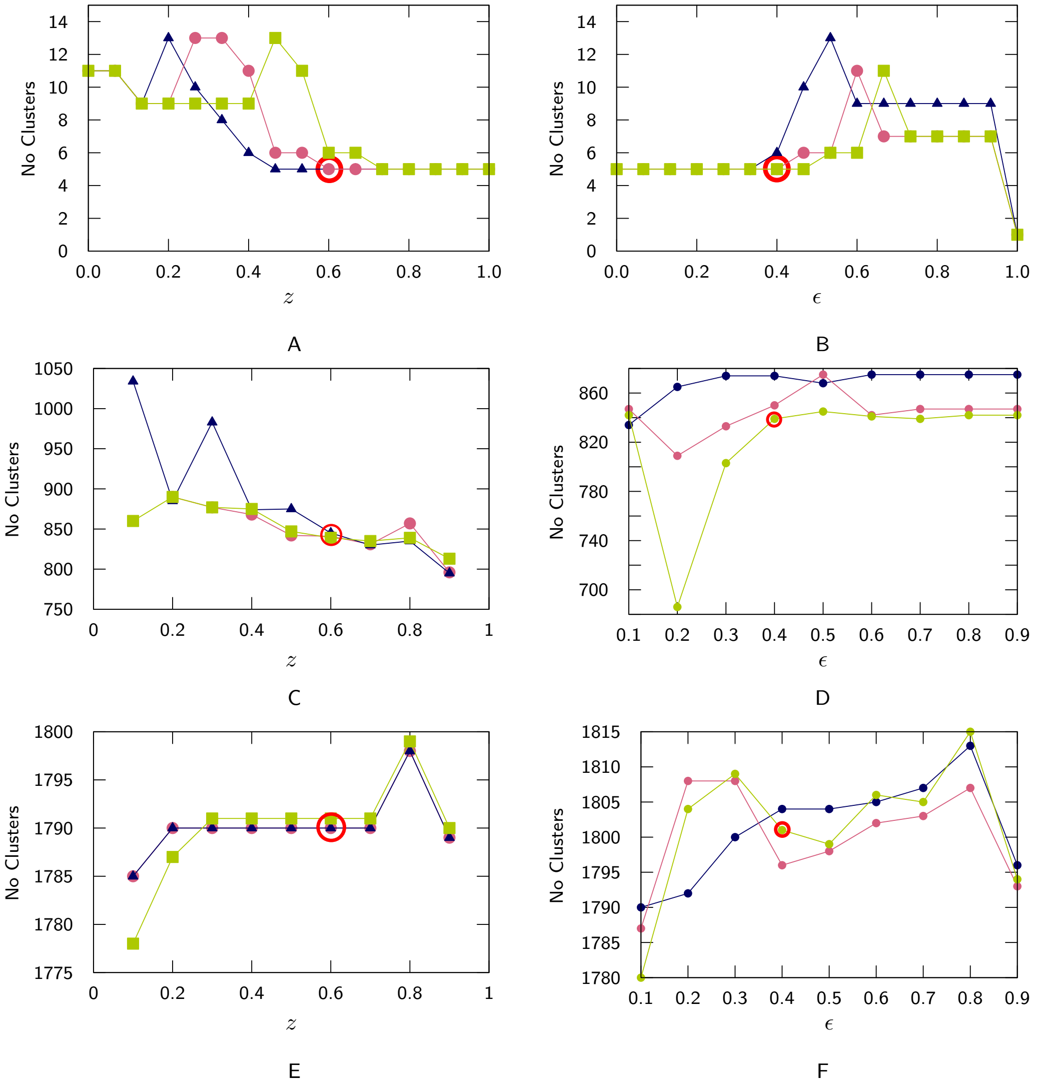

Figure S-3: **Variation of the cluster size against the z and ϵ parameters.** Variation of the number of clusters as a function of z with given ϵ (right panels) and as a function of ϵ with given z (left panels). The top panels show the Florentine families, the middle panels the *C. elegans* and the bottom panels the EU-airports multiplexes. In the left panels data are shown for $\epsilon = 0.4$ (blue triangles), $\epsilon = 0.5$ (pink circles) and $\epsilon = 0.6$ (green squares). In the right panels data are shown for $z = 0.4$ (blue triangles), $z = 0.5$ (pink circles) and $z = 0.6$ (green squares). The red circles show the values used in the main manuscript.

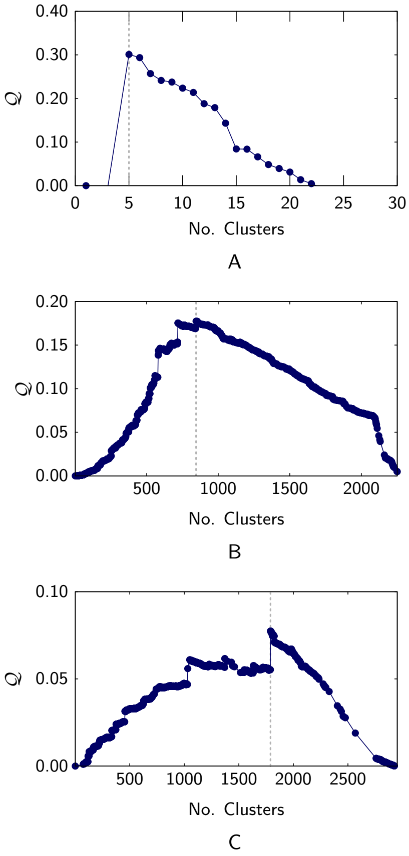

Figure S-4: **Number of clusters against the score function $\mathcal{Q}$ (link–modularity).** (A) Florentine Families Multiplex Network ($\epsilon = 0.5, z = 0.6$). (B) The Multiplex Connectome of *C. elegans* ($\epsilon = 0.4, z = 0.6$) and (C) for the European Multiplex Air Transportation Network ($\epsilon = 0.4, z = 0.6$). The maximum of $\mathcal{Q}$ determines the number of clusters which define the multlink communities of the multiplex network.

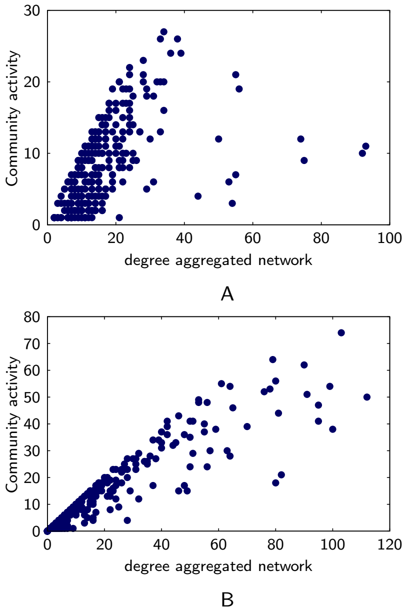

Figure S-5: **Aggregated degree vs. community activity** (A) Multiplex Connectome of *C. elegans* and (B) for the European Mutliplex Air Transportation Network.
