# Supplementary figures and images for: Multilink communities of multiplex networks
Source: PLoS One. 2018 Mar 20;13(3):e0193821. doi: 10.1371/journal.pone.0193821 (PMC5860749; doi:10.1371/journal.pone.0193821)

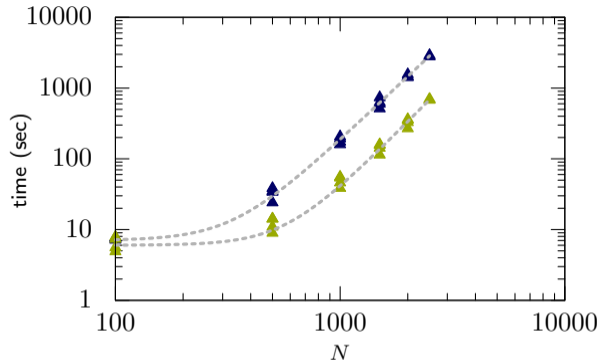

A

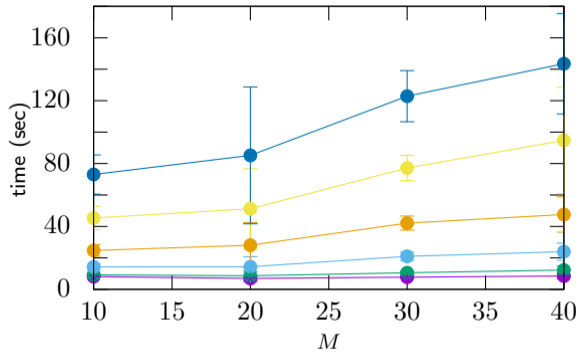

B

Supplement: S1 Fig — (A) Average CPU time elapsed to evaluate all the elements of the matrix Sik,jk as a function of the number of nodes for two families of multiplexes. Bottom line the multiplexes are defined by 〈k[α]〉 = 3 (bottom line) and 〈k[α]〉 = 3, the dashed lines show the best fit via f(N) = a + bNγ. (B) Average CPU time as a function of M. The horizontal lines correspond to multiplexes with, from bottom to top, N = 100, 500, 1000, 1500, 2000, 2500. (PDF) [file pone.0193821.s002.pdf]

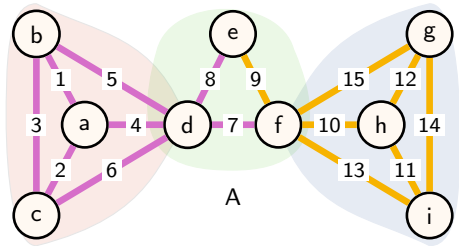

A

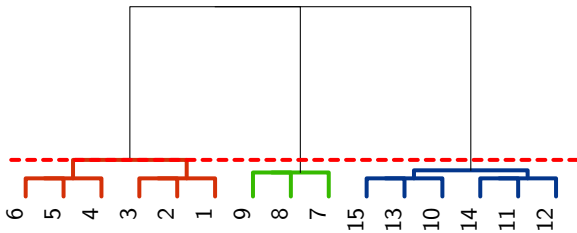

B

Supplement: S2 Fig — (A) A simple two layer multiplex network (purple and ochre links) and its multilink communities (shaded areas) and (B) its dendrogram obtained from the multilink similarity. The dashed red line shows the maximum link modularity used to define the link communities. (PDF) [file pone.0193821.s003.pdf]

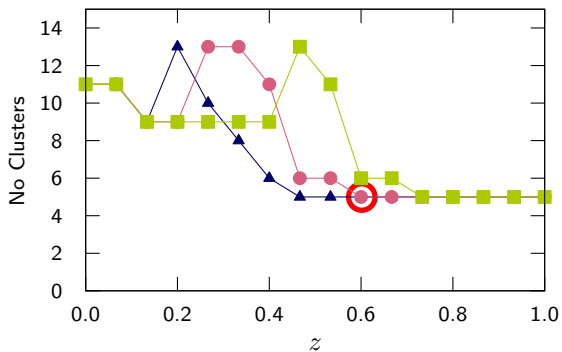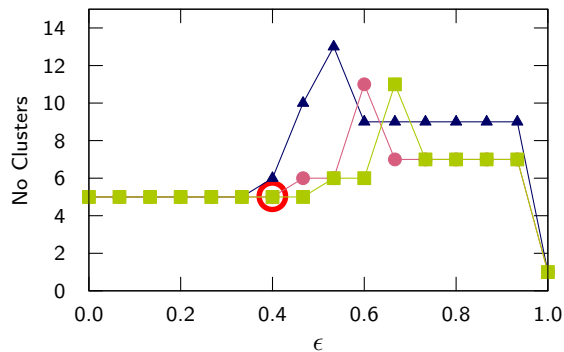

A

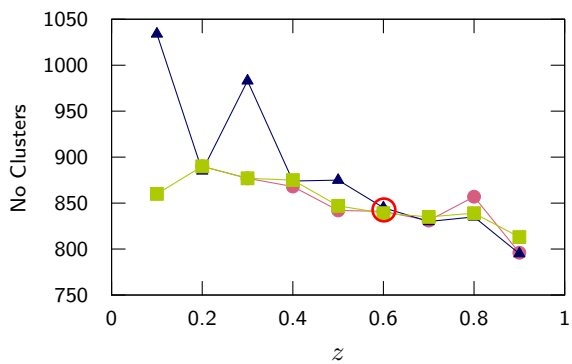

C

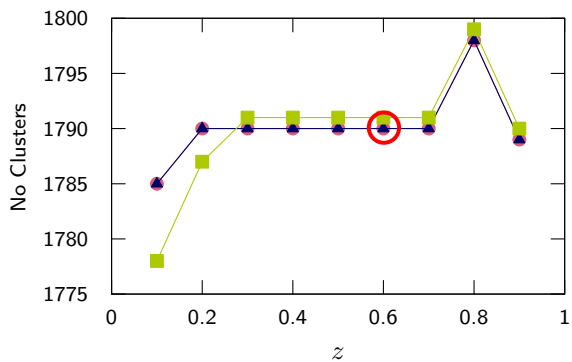

E

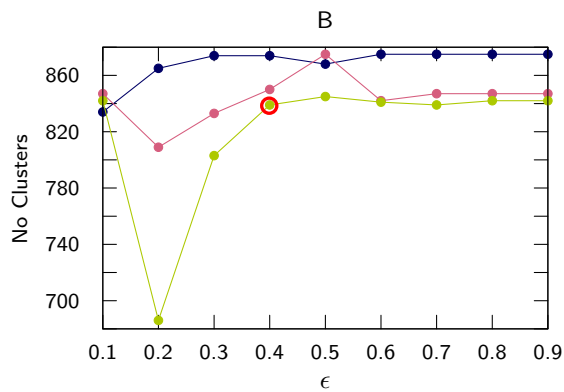

D

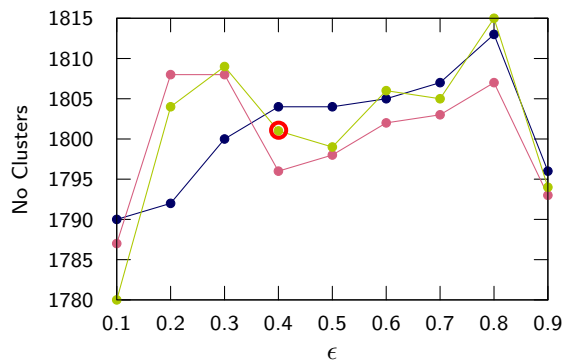

F

Supplement: S3 Fig — Variation of the number of clusters as a function of z with given ϵ (right panels) and as a function of ϵ with given z (left panels). The top panels show the Florentine families, the middle panels the C. elegans and the bottom panels the EU-airports multiplexes. In the left panels data are shown for ϵ = 0.4 (blue triangles), ϵ = 0.5 (pink circles) and ϵ = 0.6 (green squares). In the right panels data are shown for z = 0.4 (blue triangles), z = 0.5 (pink circles) and z = 0.6 (green squares). The red circles show the values used in the main manuscript. (PDF) [file pone.0193821.s004.pdf]

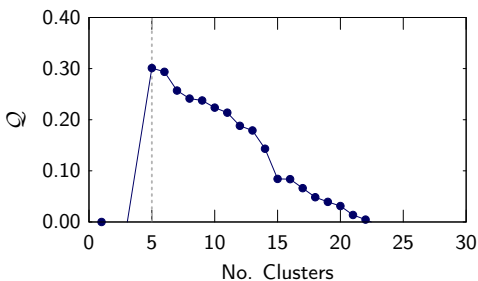

A

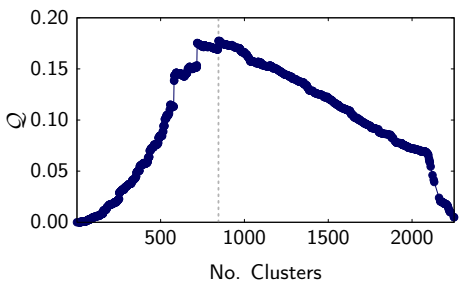

B

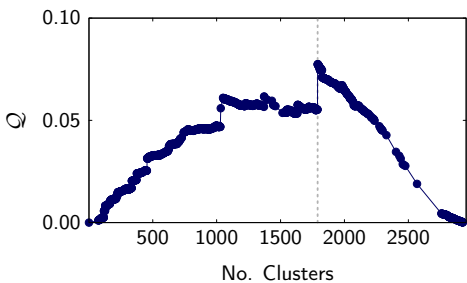

C

Supplement: S4 Fig — (A) Florentine Families Multiplex Network (ϵ = 0.5, z = 0.6). (B) The Multiplex Connectome of C. elegans (ϵ = 0.4, z = 0.6) and (C) for the European Multiplex Air Transportation Network (ϵ = 0.4, z = 0.6). The maximum of Q determines the number of clusters which define the multlink communities of the multiplex network. (PDF) [file pone.0193821.s005.pdf]

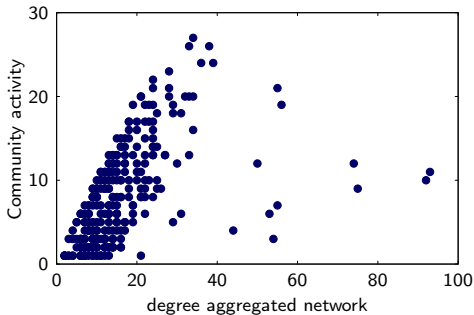

A

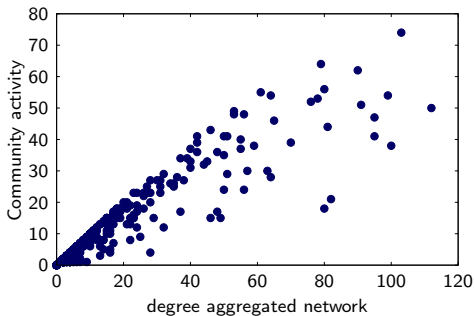

B

Supplement: S5 Fig — (A) Multiplex Connectome of C. elegans and (B) for the European Mutliplex Air Transportation Network. (PDF) [file pone.0193821.s006.pdf]
