# Supplementary material for: Multilink communities of multiplex networks
Source: PLoS One. 2018 Mar 20;13(3):e0193821. doi: 10.1371/journal.pone.0193821 (PMC5860749; doi:10.1371/journal.pone.0193821)
Supplement: S1 Data — (ZIP) [file pone.0193821.s007.zip › Padgett-Florence-Families_Multiplex_Social/README.pdf]

PADGETT-FLORENTINE-FAMILIES MULTIPLEX NETWORK

Last update: 1 July 2014

Reference and Acknowledgments

This README file accompanies the dataset representing the multiplex social network of a corporate law partnership. If you use this dataset in your work either for analysis or for visualization, you should acknowledge/cite the following paper:

```
"Robust Action and the Rise of the Medici, 1400-1434"
JF Padgett, CK Ansell
American journal of sociology, 1259-1319 (1993)
```

Description of the dataset

The multiplex social network consists of 2 layers (marriage alliances and business relationships) describing florentine families in the Renaissance.

There are 16 nodes in total, labelled with integer ID between 1 and 16, with 35 connections. The multiplex is undirected and unweighted, stored as edges list in the file

```
Padgett-Florentine-Families_multiplex.edges
```

with format

```
layerID nodeID nodeID weight
```

(Note: all weights are set to 1)

The IDs of all layers are stored in

```
Padgett-Florentine-Families_layers.txt
```

The IDs of nodes and their labels can be found in the file

```
Padgett-Florentine-Families_nodes.txt
```

License

The PADGETT-FLORENTINE-FAMILIES MULTIPLEX DATASET is provided "as is" and without warranties as to performance or quality or any other warranties whether expressed or implied.
