# Supplementary material for: Multilink communities of multiplex networks
Source: PLoS One. 2018 Mar 20;13(3):e0193821. doi: 10.1371/journal.pone.0193821 (PMC5860749; doi:10.1371/journal.pone.0193821)
Supplement: S2 Data — (ZIP) [file pone.0193821.s008.zip › EUAir_Multiplex_Transport/README.pdf]

EU-AIR-TRANSPORTATION MULTIPLEX NETWORK

Last update: 1 July 2014

Reference and Acknowledgments

This README file accompanies the dataset representing the multiplex air transportation network of Europe.
If you use this dataset in your work either for analysis or for visualization, you should acknowledge/cite the following paper:

```
“Emergence of network features from multiplexity”
Alessio Cardillo et al
Scientific Reports 3, 1344 (2013)
```

that can be found at the following URL:

http://www.nature.com/srep/2013/130227/srep01344/full/srep01344.html

See the official webpage of this dataset

http://complex.unizar.es/~atnmultiplex/

for further details.

Description of the dataset

The multilayer network is composed by thirty-seven different layers each one corresponding to a different airline operating in Europe.

There are 450 nodes in total, labelled with integer ID between 1 and 450, with 3588 connections. The multiplex is undirected (with only one direction specified) and unweighted, stored as edges list in the file

```
EUAirTransportation_multiplex.edges
```

with format

```
layerID nodeID nodeID weight
```

(Note: all weights are set to 1)

The IDs of all layers are stored in

```
EUAirTransportation_layers.txt
```

The IDs of nodes, together with their geographical coordinates (latitude and longitude in deg) can be found in the file

```
EUAirTransportation_nodes.txt
```

License

The EU-AIR-TRANSPORTATION MULTIPLEX DATASET is provided "as is" and without warranties as to performance or quality or any other warranties whether expressed or implied. By using this data set, you agree not to perform reverse engineering to extract airlines, airport names, or any other information, and not to perform comparative studies about the performance of different air transport actors.

Copyright (C) 2013 Cardillo, Gomez-Gardenes and Zanin.

Permission is granted to copy, distribute and/or modify this document under the terms of the GNU Free Documentation License, Version 1.3 or any later version published by the Free Software Foundation; with no Invariant Sections, no Front-Cover Texts, and no Back-Cover Texts. A copy of the license is included in the section entitled "GNU Free Documentation License".

You can find a copy of this license here: http://www.gnu.org/copyleft/fdl.html

Contacts

If you find any error in the dataset or you have questions, please contact

```
Alessio Cardillo
```

web: http://bifi.es/~cardillo/
